# Exploring the Therapeutic Potential of Gene Therapy in Arrhythmogenic Right Ventricular Cardiomyopathy

**DOI:** 10.3390/biomedicines12061351

**Published:** 2024-06-18

**Authors:** Juan Mundisugih, Dhanya Ravindran, Eddy Kizana

**Affiliations:** 1 Centre for Heart Research, Westmead Institute for Medical Research, Westmead, NSW 2145, Australia; 2 Department of Cardiology, Westmead Hospital, Westmead, NSW 2145, Australia; 3 Sydney Medical School, The University of Sydney, Camperdown, NSW 2050, Australia

**Keywords:** adeno-associated virus, gene therapy, arrhythmogenic right ventricular cardiomyopathy, genetic heart disease, biotechnology

## Abstract

Right dominant arrhythmogenic cardiomyopathy, commonly known as Arrhythmogenic Right Ventricular Cardiomyopathy (ARVC), represents a formidable challenge in cardiovascular medicine, as conventional therapies are commonly ineffective in impeding disease progression and the development of end-stage heart failure. Recombinant adeno-associated virus (AAV)-mediated gene therapy presents a promising avenue for targeted therapeutic interventions, potentially revolutionising treatment approaches for ARVC patients. Encouraging results from preclinical studies have sparked optimism about the possibility of curing specific subtypes of ARVC in the near future. This narrative review delves into the dynamic landscape of genetic therapy for ARVC, elucidating its underlying mechanisms and developmental stages, and providing updates on forthcoming trials. Additionally, it examines the hurdles and complexities impeding the successful translation of ARVC genetic therapies into clinical practice. Despite notable scientific advancements, the journey towards implementing genetic therapies for ARVC patients in real-world clinical settings is still in its early phases.

## 1. Introduction

Arrhythmogenic cardiomyopathy (ACM) is a progressive disorder affecting the cardiac muscle, characterised by arrhythmias that are not related to ischemia, hypertension, or valvular heart disease [1]. ACM can manifest as either left or right dominant, with the latter commonly known as Arrhythmogenic Right Ventricular Cardiomyopathy (ARVC). Classic ARVC primarily stems from genetic variants in the *PKP2*, *JUP*, *DSG2*, and *DSC2* genes, responsible for encoding plakophilin-2, plakoglobin, desmoglein-2, and desmocollin-2, respectively [2]. Carriers of variants in the *DSP* gene, encoding desmoplakin, typically present with left-dominant ACM but may also exhibit symptoms of ARVC [2]. These proteins collectively form the desmosome, a specialised structure within the intercalated disc. They play a crucial role in providing structural stability and enabling inter-cellular communication. Mutations in these genes result in structural changes leading to fibrosis and inflammation, contributing to the arrhythmogenic substrate [3]. There are also pathogenic variants in non-desmosomal proteins, such as transmembrane protein 43 (*TMEM43*), phospholamban (*PLN*), and desmin (*DES*), which represent rare causes of ARVC [4]. *PKP2*, *JUP*, *DSG2*, *DSC2*, *DSP*, *TMEM43*, *PLN*, and *DES* are associated with autosomal dominant ARVC, except for *DSC2* and *DSG2*, which are associated with both autosomal dominant and, rarely, autosomal recessive ARVC [5].

The prevalence of ARVC is estimated to range between 1 in 2000 and 1 in 5000, varying with geographic location, and with a slight male predominance (approximately 55%-60%) [6]. Penetrance of ARVC appears to increase with age, with most cases diagnosed between the second and fourth decades of life [6]. The disease manifests an elevated risk of life-threatening arrhythmias, alongside loss of myocardial mass and the presence of fibrofatty infiltrates [6]. Approximately 10% of cases are diagnosed following episodes of resuscitated cardiac arrest or sudden cardiac death (SCD) [7], highlighting the severity of its clinical manifestations. It is proposed that ventricular fibrillation typically occurs in the early stages of ARVC, while ventricular tachycardia tends to emerge later in the disease progression, often due to scar tissue development. Heart failure (HF) at initial diagnosis is uncommon, and the emergence of HF symptoms typically indicates an advanced stage of the disease [8].

Current management strategies, which include risk stratification, medications, implantable cardioverter defibrillators, and catheter ablation, are limited to symptomatic relief, lacking proven disease-modifying effects. Consequently, many patients progress to end-stage HF, necessitating cardiac transplantation to avert mortality. Given the challenging prognosis of ARVC, innovative therapies are imperative. Gene therapy has emerged as a promising novel strategy that involves the genetic alteration of cells, necessitating vectors to transport genetic material into various cells, tissues, and organs. These vectors are broadly categorised as viral vectors, non-viral vectors, and engineered vectors. Among vector systems, viral vectors, particularly the recombinant adeno-associated virus (rAAV) system [9,10], have emerged as a preferred and efficient tool for facilitating gene therapy, as the currently available non-viral or engineered vectors do not meet the ideal vector properties. Despite initial setbacks witnessed in the CUPID trial [11], positive results from clinical trials involving *LAMP2B* for Danon disease [12] and protein phosphatase inhibitor-1 for advanced HF signify ongoing progress in this field [13]. This review aims to provide a comprehensive evaluation of gene therapy’s potential as a curative treatment for ARVC, including its underlying mechanisms, stages of development, and impending challenges.

## 2. Gene Therapy: Understanding the Fundamentals

The identification of DNA as the biomolecule responsible for genetically inherited diseases and the prospect of tangible therapeutic benefits to patients was originally introduced over 40 years ago [14]. It is now evident that gene therapy holds a broader potential, extending to acquired polygenic diseases such as ischemic heart disease, arrhythmias, and HF. Advances in understanding the molecular mechanisms underlying these diseases, coupled with the refinement of gene transfer technology, has propelled certain cardiovascular diseases closer to the possibility of gene-based therapy as a promising alternative to existing treatments [15].

For decades, gene therapy technology has utilised the natural ability of viruses to target cells and deliver transgenes. The prevalent utilisation of viral vector systems in preclinical models of gene therapy underscores the heightened gene transfer efficiencies attainable through these systems. Currently, the viral vectors showing greatest efficiency in preclinical and clinical investigations are derived from retroviruses, lentiviruses, adenoviruses, and AAV [16]. Among these viral vectors, the rAAV vector system is regarded as the lead candidate for cardiac gene transfer, and is hence the most extensively investigated vehicle for clinical gene therapy. The characteristics of rAAV that render it favourable as a vector include high transduction efficiency, low immunogenicity, unique capsid-dependent tissue tropism, and the capacity to confer long-term expression without significant genomic integration [9,10].

**Figure 1:**
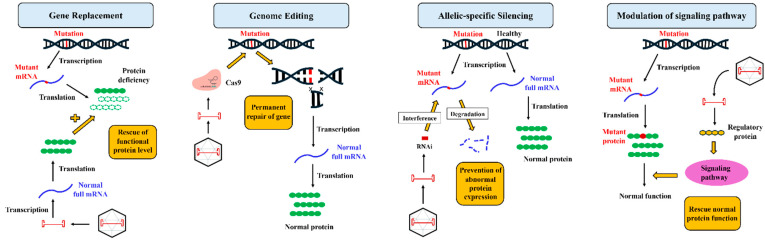
Four Main Strategies of Gene Therapy.

Gene therapy encompasses various molecular strategies tailored to the molecular pathophysiology of the target disease, as illustrated in Figure 1. These strategies include gene replacement, which is utilised in conditions of protein deficiency by introducing functional gene copies to ameliorate or replace defective proteins. Secondly, genome editing, exemplified by CRISPR/Cas9, allows precise modification of DNA sequences but faces challenges such as off-target effects [17]. Allelic-specific silencing employs RNA interference to selectively suppress mutated alleles, while sparing healthy ones [18]. Lastly, the modulation of signalling pathways targets disease mechanisms akin to small molecule therapies but offers broader targeting possibilities [19]. These diverse modalities of gene therapy hold promise for transformative treatments by offering multiple options to address different aspects of disease mechanisms, which proves particularly beneficial in conditions with multifactorial origins, such as ARVC.

## 3. Gene Therapy Breakthroughs in ARVC Treatment

### 3.1. Plakophilin-2 (PKP2) Pathogenic Variant

The *PKP2* gene has been the primary focus of preclinical studies due to its prevalence in clinical cohorts. In the initial in vitro investigation by Inoue et al. [20], an induced pluripotent stem cell-derived cardiomyocytes (iPSC-CMs) model was generated from an ARVC patient harbouring a heterozygous frameshift mutation in the *PKP2* gene. Following monolayer differentiation, a deficiency of PKP2 protein resulted in diminished contractility, disrupted intercalated disc structures, and impaired desmosome assembly in iPSC-CMs. To explore the feasibility of gene replacement therapy in human cells, a rAAV2 containing a full sequence of the human *PKP2* sequence with a strong but non-cell-specific cytomegalovirus (CMV) promoter was engineered. Remarkably, the AAV-mediated gene replacement of *PKP2* successfully restored contractility and reinstated desmosome assembly, as evidenced by subsequent desmosome imaging. In recent preclinical studies, Wu et al. [21] and Kyriakopoulou et al. [22] also reported similar results, with the latter study demonstrating that AAV6-PKP2 gene replacement not only restored desmosomal proteins but also enhanced sodium-dependent conduction in an iPSC-CMs model, indicating the rescue of the arrhythmogenic substrate. To closely mimic the human ARVC model, Kyriakopoulou et al. additionally developed three-dimensional (3D)-engineered human myocardium consisting of 70% iPSC-CMs and 30% human foreskin fibroblasts embedded in a collagen type 1-based hydrogel. This model offers a more mature cardiomyocyte phenotype compared to traditional 2D cell cultures, while facilitating the sequential evaluation of contractile properties at different stages of tissue maturation [23]. In line with the iPSC-CM models, they observed that AAV6-PKP2 gene replacement yielded both molecular and functional improvements in the 3D model. These pivotal results laid the groundwork for subsequent in vivo investigations.

Wu et al. [21] and Kyriakopoulou et al. [22] extended their research into an in vivo model to enhance its clinical relevance. Wu et al. [21] utilised a well-characterised murine model with cardiomyocyte-specific tamoxifen (TAM)-activated *Pkp2* knockout (*Pkp2*-cKO), and engineered two distinct AAV9 vectors expressing either human *PKP2* (TN-401) or its mouse ortholog (AAV9:mPkp2). Initially, they administered a single systemic dose of the viral vector three weeks before tamoxifen (TAM) injection, thus preceding the onset of disease. Remarkably, both human *PKP2* and the mouse ortholog were found to effectively prevent detrimental cardiac manifestations and improve survival outcomes in *Pkp2*-cKO mice when delivered via the AAV9 vector. Subsequently, in dose–response assessments of TN-401 or AAV9:mPkp2, *Pkp2*-cKO mice received single systemic treatments at various doses one-week post-TAM induction. Notably, dose escalation studies revealed efficacy at doses equal to or exceeding 3 × 10^13^ vg/kg, exhibiting the prevention of adverse right ventricular remodelling, enhanced ventricular function, reduced fibrosis, and improved electrophysiological properties. Moreover, to explore the potential of PKP2 restoration after overt structural changes indicative of ARVC progression, animals were dosed with AAV9:mPkp2 via retro-orbital injection at 1 × 10^14^ vg/kg, initiated 2.5 weeks post-cardiac *Pkp2* deletion. Encouragingly, this therapeutic approach significantly extended the median lifespan by ≥50 weeks during the one-year follow-up, paralleling the survival benefits observed in the preventive treatment mode. Nonetheless, the murine *Pkp2*-cKO model utilised in this study is imperfect, displaying a more pronounced manifestation than the haploinsufficiency observed in humans. As a result, the therapeutic dosage that effectively alleviates the disease phenotype in this model may vary significantly from what is needed for humans. In this study, the model exhibited accelerated disease progression, culminating in death between 3 and 6 weeks after TAM injection, presenting a challenge in establishing correlations with disease stages in humans.

Unlike the study conducted by Wu et al. [21], Kyriakopoulou et al. [22] utilised an improved mouse model of ACM that closely resembles the human pathogenic *PKP2c.2013delC* variant, exhibiting a significant decrease in levels of cardiac desmosomal and adherens junctional proteins [24]. In their in vivo studies, they employed cardiotropic recombinant AAV9 vectors and the CMV promoter to express murine wild-type *Pkp2*. To enable detection of the exogenously delivered PKP2, they fused the murine wild-type *Pkp2* with a MYC epitope. Initially, as a proof of concept, they administered AAV9-PKP2 to 5-day-old *Pkp2* mutant pups, revealing that a high dose of the AAV9 vector could transduce up to 76% of cardiac myocytes. Given that ARVC symptoms often manifest during early adulthood, they investigated the effects of AAV9-PKP2 administration at a more clinically relevant stage. Intravenously injecting Pkp2 mutants at 2 months of age with either AAV9-PKP2 or AAV9–control, they observed that the treatment resulted in the restoration of not only PKP2 but also other proteins such as plakoglobin, desmoplakin, and desmoglein-2 to the desmosome. These improvements in PKP2 levels and other junctional proteins were accompanied by the preservation of cardiac function. Remarkably, the therapeutic effects of this treatment persisted for up to 10 months following a single injection. However, their data indicated a moderate expression of exogenous PKP2 in the mice liver, despite minimal levels elsewhere. This implies that an additional refinement of the AAV vector may be required before considering its use in humans, to prevent potential unintended effects, even if the therapeutic results in the heart are promising.

Bradford et al. [25] optimised their vector by employing a cardiotropic rAAV9 vector coupled with a cardiac-selective human Troponin T (cTnT) promoter to drive wild-type mouse *Pkp2* expression. In contrast to the study conducted by Kyriakopoulou et al. [22], they utilised a genetically modified mouse carrying a mutation equivalent to the human splice site variant *IVS10-1 G > C*. This mutation holds significant clinical relevance, as it stands as one of the most common PKP2 mutations in ARVC cohorts, according to the Atlas of Cardiac Genetic Variation [26]. Their model effectively exhibited several important human ARVC features, including sudden cardiac death, ventricular arrhythmia, biventricular dysfunction, desmosomal abnormalities, inflammation, and fibrofatty replacement of the myocardium. Their AAV-PKP2 strategies successfully restored PKP2 protein expression in the early stages of ARVC development, mitigating not only the pronounced cardiac desmosomal disruption but also addressing gap junction deficits that collectively contribute to the arrhythmia and biventricular dysfunction in ARVC. Their longitudinal late-stage administration studies further demonstrated that AAV-PKP2 could reverse multiple histopathological cardiac abnormalities, such as reducing cardiac fibrosis, fat deposition, inflammation, and cytokine production, alongside improving left and right ventricular dimensions and function, and alleviating cardiac arrhythmias. While they did not examine exogenous PKP2 levels in other organs, they evaluated liver function by measuring circulating levels of the liver enzymes alkaline phosphatase (ALP) and alanine aminotransferase (ALT). They observed no significant differences between wild-type controls and AAV-PKP2-treated genetically modified mice at 6 months of age. This suggests that, if desired, detrimental effects on other organs can be circumvented by employing a cardiotropic capsid and a cardiac-specific promoter to ensure sustained gene expression preferentially within the heart.

The study conducted by van Opbergen et al. [27] further reinforces the importance of careful vector design in AAV-mediated gene therapy for ARVC. To achieve an enhanced targeting of the heart, they utilised an rAAVrh.74 vector, recognised for its preferential binding to striated muscle, in conjunction with the cardiac-specific cTnT promoter to control expression of the human *PKP2* transcript variant A (*hPKP2a*). The viral vector was administered systemically to TAM-activated *Pkp2*-cKO murine models at two distinct time points. In the first cohort, the viral vector was administered before the onset of disease, followed by TAM injection 28 days later. In the second group, treatment with rAAVrh.74-hPKP2a occurred either 7 or 14 days after TAM injection, corresponding to disease onset. In the pre-disease treatment group, rAAVrh.74-hPKP2a gene therapy resulted in a dose-dependent reduction in fibrosis, preservation of contractile function in the left ventricle (LV), and mitigation of right ventricle (RV) enlargement. Strikingly, treatment initiated after disease onset effectively halted disease progression, including arrhythmias and cardiomyopathy, and remarkably prolonged survival. Their approach employing the rAAVrh.74 vector and cTnT promoter demonstrated robust transduction efficiency and an elevated expression of hPKP2a specifically within the heart, with minimal expression observed in skeletal muscle and the liver (less than 300-fold compared to the heart). These results highlight the significance of selecting the appropriate capsid and promoter to guarantee successful expression of the gene of interest specifically within the heart, while reducing expression in other tissues.

**Figure 2:**
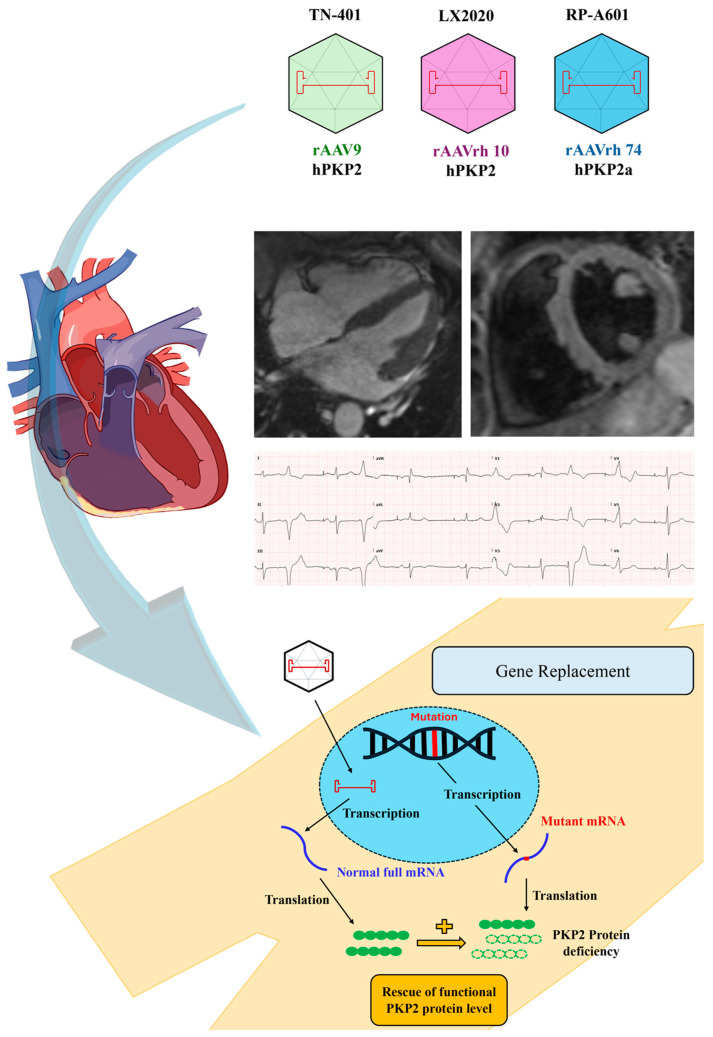
FDA-approved AAV-mediated Gene Therapies for Individuals Diagnosed with ARVC According to The 2010 Revised Task Force Criteria. These therapies are indicated for patients with confirmed genetic testing showing a pathogenic variant in PKP2 and a high frequency of premature ventricular complexes.

Regardless of variations in vector designs and model systems, the studies conducted by Wu et al. [21], Kyriakopoulou et al. [22], Bradford et al. [25], and van Opbergen et al. [27] collectively emphasise the potential of AAV-mediated gene therapy to mitigate the advancement of *PKP2*-associated ARVC and improve cardiac function in preclinical models. To date, three AAV-mediated gene therapies for *PKP2*-associated ARVC have received approval from the US FDA to proceed into phase 1 clinical trials (Figure 2). Among these is TN-401, a variant of rAAV9-hPKP2 used in the study by Wu et al. [21]. The other approved products include LX2020, which utilises the rAAVrh.10-hPKP2 vector developed by Lexeo Therapeutics Inc. (United States), and RP-A601, the therapeutic agent utilised in the study conducted by van Opbergen et al. [27], employing rAAVrh.74-hPKP2a. This advancement toward regulatory approval underscores the growing acknowledgment and validation of AAV-mediated gene therapy as a promising and innovative treatment approach for cardiac genetic disorders with previously guarded prognoses.

### 3.2. Phospholamban (PLN) Pathogenic Variant

The *PLN* mutation (*PLN-R14del*) has attracted considerable attention due to its increasing prevalence among patients worldwide. Particularly in the Netherlands, this genetic variant stands out as the most common mutation associated with cardiomyopathies, accounting for approximately 12% and 15% of patients diagnosed with ACM and dilated cardiomyopathy (DCM), respectively [28]. Initial investigations into gene therapy targeting *PLN-R14del* involved the use of iPSC-CMs from a patient carrying this mutation. Karakikes et al. [29] discovered that the *PLN-R14del* mutation triggers abnormalities in calcium handling, electrical instability, and the abnormal cytoplasmic distribution of PLN protein in iPSC-CMs. To correct PLN expression in this iPSC-CM model, they developed an AAV type 6 vector capable of downregulating endogenous *PLN* using an intronic artificial microRNA (miR-PLN), while simultaneously facilitating the overexpression of a microRNA (miRNA)-resistant, codon-optimised *PLN*. These vectors enabled a robust expression of wild-type *PLN*, alongside an approximately 50% reduction in endogenous mutant *PLN* transcripts. Seven days following gene therapy, it was observed that the AAV6-mediated overexpression of wild-type *PLN* reduced the occurrence of arrhythmogenic episodes compared to non-transduced cells.

Subsequently, Dave et al. [30] presented promising findings from their study on AAV9-CRISPR/Cas9 gene editing targeting the *PLN-R14del allele*. They initiated their research by creating humanised mice expressing the mutant *PLN* (*hPLN-R14del*) in a heterozygous state, mirroring the early phase of the disease phenotype in human carriers. This phase is characterised by a risk of arrhythmias without evident structural heart remodelling. Two-month-old heterozygous *hPLN-R14del* mice then received an AAV9/CRISPR-Cas9-gRNA vector via tail vein injection. This approach led to a reduction in mutant PLN transcript levels and consequent decrease in mutant PLN protein expression, thereby preserving the function of the wild-type PLN. Eight weeks post-treatment, mice treated with gene therapy exhibited significantly reduced right and left end-diastolic volumes and stroke volumes, along with a decreased susceptibility to ventricular arrhythmia. These findings provide preclinical evidence supporting potentially translatable gene-editing strategies to mitigate the arrhythmogenic phenotype in human patients with *PLN-R14del* disease. However, rigorous preclinical testing, addressing safety concerns, and navigating regulatory processes remain indispensable stages in advancing from bench to bedside in the translational journey.

### 3.3. Desmoglein-2 (DSG2) Pathogenic Variant

In contrast to the *PKP2* and *PLN* pathogenic variants, certain ARVC pathogenic variants, or at least DSG2 deficiency [31], might be concealed among genetically undiagnosed idiopathic DCM patients with severe HF. Shiba et al. [31] identified a case of DSG2-deficient cardiomyopathy resulting from a homozygous stop-gain mutation, which was initially diagnosed as idiopathic DCM. They observed disrupted intercalated discs and an abnormal deposition of desmosome proteins in vacuolated structures in the patient’s myocardium, characteristics of ARVC. They subsequently generated an iPSC-CMs model from this patient, revealing abnormal excitation, increased arrhythmias, a disturbed conduction velocity, tissue fragility, and weakened contraction force. Remarkably, these abnormalities were successfully rectified by AAV2-mediated gene replacement of *DSG2*. Their results suggest that gene replacement therapy aimed at restoring *DSG2* before disease progression could serve as an effective upstream therapy, potentially preventing the transition to advanced HF. However, their 2D tissue model represents a controlled environment distinct from the complex pathological substrates encountered in *DSG2*-associated ARVC. The assumption of uniform transduction and *DSG2* expression in all cardiomyocytes in vitro may not hold true in vivo, given the variable transduction efficiencies and levels of gene expression associated with heterogenous vector delivery. Hence, further in vivo assessments will be imperative to assess this otherwise promising therapeutic strategy. Renovacor, Inc., based in the United States, has actively expanded its pipeline to advance their AAV gene therapy program. This initiative aims to develop a precision therapy targeting the most significant genetic segments of arrhythmogenic cardiomyopathy, including those with the *DSG2* pathogenic variant [32].

## 4. Challenges in Translation from Bench to Bedside

**Figure 3:**
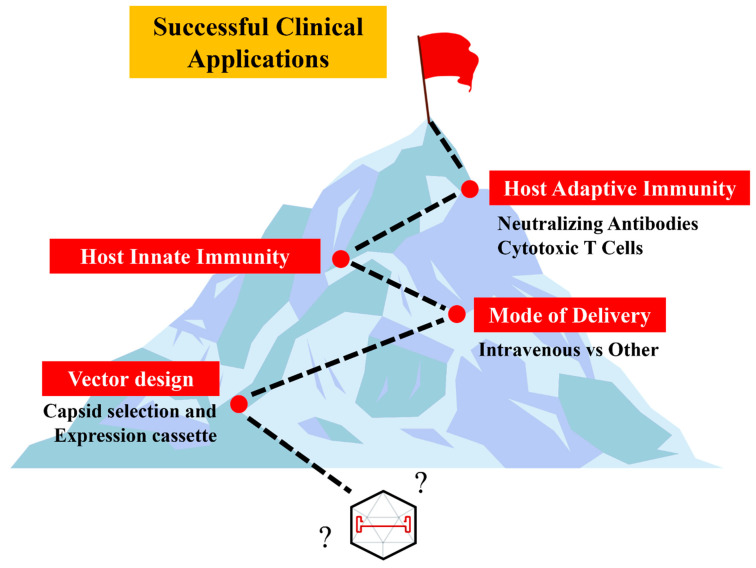
AAV Translational Challenges to Successful Clinical Applications.

There is considerable excitement surrounding the translation of these proof-of-concept studies into clinical applications. Yet, there are still many significant challenges that must be addressed for successful applications (Figure 3). It is important to note that the success of gene therapy relies on effective and safe gene transduction, which is largely influenced by rAAV vector design, mode of delivery, and vector–host immune system interactions.

### 4.1. Recombinant AAV Vector Design

The AAV capsid, forming the outer protein shell, plays a crucial role in determining vector tropism and in regulating the effectiveness of AAV receptor-mediated cellular entry and transgene expression [33]. Variations in the amino acid sequences of capsids can result in alterations in tropism across different AAV serotypes [34]. Three FDA-approved AAV-mediated gene therapies for *PKP2*-associated ARVC utilised naturally occurring AAV9, AAVrh.10, and AAVrh.74 serotypes. Despite differences in their capsid protein sequences and receptors, they exhibit preferential binding to striated muscle and have demonstrated success in cardiac transduction in preclinical studies. However, extrapolating these results directly to humans is challenging, due to the discrepancy in AAV’s ability to target specific tissues across different species [35]. This variability is evident in the CUPID trial, where AAV1 initially showed promising outcomes in preclinical and early-phase clinical trials [36]. However, the results from the phase 2b trial did not demonstrate any improvement in hospital admission or mortality related to heart failure in patients with moderate to severe heart failure who were treated with AAV1/sarco(endo)plasmic reticulum Ca^2+^ ATPase 2a isoform (SERCA2a). These disappointing outcomes are believed to be due to poor transduction efficiency [11]. Consequently, various strategies have been employed to develop novel rAAVs with the aim of achieving improved tropism in humans. These strategies include post-translational modifications through specific mutations, the creation of chimeric capsids, peptide and protein insertions, capsid shuffling, de novo mutations, peptide libraries, chemical conjugation, partner biomolecules, and transcapsidation [37]. One notable outcome of these efforts is the creation of a chimeric AAV2/AAV8 vector (known as AAV2i8) [38], currently under investigation in a clinical trial focusing on protein phosphatase inhibitor-1 for advanced HF [13]. Preliminary findings suggest that the AAV2i8 vector’s capsid demonstrates a pronounced affinity for cardiac tissue when administered intra-coronary at relatively low doses in human patients [13]. This effective transduction resulted in clinically meaningful improvements in the left ventricular ejection fraction (LVEF), NYHA Functional Class (NYHA FC), Minnesota Living with Heart Failure Questionnaire (MLHFQ), cardiopulmonary exercise test (VO_2_ max), and 6 min walk test (6MWT) at the 12-month follow-up. The ability to achieve a high transduction with relatively lower doses also bodes well for reducing vector-related toxicity and vector manufacturing.

In addition, the expression cassette design including promoter and other regulatory elements within an rAAV vector genome plays a pivotal role in governing the expression of therapeutic genes, significantly impacting both the extent and specificity of gene expression. In diseases such as ARVC, localisation of gene expression to the ventricular myocardium is crucial to prevent off-target effects in non-cardiac tissues. Hence, the cTnT promoter was selected for FDA-approved AAV-mediated gene therapies targeting *PKP2*-associated ARVC. However, compared to robust promoters like CMV, the cTnT promoter may yield lower levels of transgene expression, potentially limiting its efficacy if high expression levels are required to address ARVC phenotypes in humans. One potential solution to this issue involves incorporating enhancer sequences, which have the capability to enhance expression activity through either integration with the promoter or by providing specificity. Encouraging findings have emerged regarding candidate enhancer elements from the *LMNA* and *MYH7* genes, which exhibit expression in the heart and are upregulated in cardiomyopathy [39]. These enhancers might enable disease-triggered transcription, elevating transgene expression solely in pathological contexts. Further research is needed to explore the suitability of potential enhancers for *PKP2*-ARVC. Lastly, another approach to improve tissue specificity involves detargeting transgene expression from specific organs using endogenously expressed miRNAs, thereby preventing unintended transgene expression in non-target cells. For instance, incorporating liver-specific miR-122 target sequences (*miR-122T*) into the 3′-untranslated region (UTR) of the cassette significantly reduced liver expression [40].

### 4.2. Mode of AAV Delivery

Systemic AAV delivery has been employed in all preclinical studies conducted for ARVC, as discussed earlier. The recently approved phase 1 clinical trials of FDA-approved AAV-mediated gene therapies targeting *PKP2*-associated ARVC (TN-401: NCT06228924, LX2020: NCT06109181, RP-A601: NCT05885412) are set to administer the gene therapy product intravenously. While systemic or intravenous delivery of AAV offers non-invasiveness and ease of administration, careful consideration must be given to potential off-target effects, immune responses, and dose limitations. Distributing a viral vector through the bloodstream will disseminate the virus to unintended tissues or organs. This not only raises safety concerns but also diminishes the amount of vector available for delivery to the intended targets, necessitating higher total doses for systemic therapy [41]. With such an increased dose of administration, the total capsid protein load in a typical systemic dose is 50–100 times that of a typical childhood immunisation, hence eliciting a strong immune reaction, and the need for immunosuppression is unsurprising [42]. A higher total capsid load increases the likelihood of immunotoxicity, particularly where capsid exposure and biodistribution are most prominent, such as in the liver [42]. Given that ARVC primarily affects the heart, intra-coronary administration may offer a more suitable approach. As evidenced by preliminary findings from clinical trials focusing on protein phosphatase inhibitor-1 for advanced HF [13], combining cardiotropic AAV with intra-coronary delivery enables an efficient and safe gene transduction with relatively low AAV doses. Importantly, this approach leads to meaningful clinical improvement, as described above, without the need of immunosuppression. Likewise, the ongoing MUSIC-HFrEF1 trial, which assess the safety and effectiveness of gene therapy targeting the modulation of SERCA2a in patients with HFrEF (heart failure with reduced ejection fraction) and NYHA class III/IV, is administering a smaller dose of AAV through intra-coronary delivery, compared to systemic administration, without immunosuppression [43]. Their initial findings indicated no gene therapy or procedure-related adverse events during the first 6 to 12 months post-infusion. Three out of the four patients showed improvements in NYHA Functional Class from Class 3 before treatment to Class 2 at both 6 and 12 months. There were clinically significant improvements in LVEF and 6MWT, decreases in Pro-BNP levels, and reductions in troponin levels from baseline to 12 months post-treatment [44].

### 4.3. Host Immune System

The host immune system has posed a persistent challenge in the gene therapy field from its inception. Despite the generally low immunogenicity of AAV vectors [9], they have demonstrated the capability to evoke notable innate and adaptive immune responses when given at a higher dose [45], which can have significant implications for both gene transduction efficiency and safety. Several studies have documented instances of innate immune responses triggered by AAVs across various animal models [46]. Of particular concern is the administration of systemic AAV-mediated gene therapy at high doses, typically ranging from 1 × 10^13^ to 2 × 10^14^ vg/kg, as anticipated in phase 1 clinical trials of FDA-approved AAV-mediated gene therapies targeting *PKP2*-associated ARVC (TN-401: NCT06228924, LX2020: NCT06109181, RP-A601: NCT05885412). Administering such high doses of AAV carries the potential risk of triggering significant innate immune responses [47]. Unfortunately, a recent incident underscores the seriousness of such risks, as a patient with Duchenne muscular dystrophy, treated with a high dose of AAV9 (1 × 10^14^ vg/kg), experienced an unexpected death from a fatal innate immune reaction, despite receiving adequate immunosuppression [48]. In light of this incident, the safety of a systemic high dose of AAVrh.10 and AAVrh.74 in human subjects requires further validation.

Apart from triggering innate immune responses, AAV vectors have been observed to also provoke adaptive humoral and cell-mediated immune responses, adding another layer of complexity to their application in gene therapy. Wild-type AAV infection is highly prevalent, and depending on the serotype and potential cross-reactivity between serotypes, a considerable proportion of patients (40–60%) may harbor pre-existing neutralising antibodies, rendering them ineligible for treatment with specific therapeutic products [49]. To date, the primary strategy employed in clinical trials (including TN-401: NCT06228924 and RP-A601: NCT05885412) to circumvent vector neutralisation by anti-AAV antibodies has been the exclusion of seropositive individuals from enrolment, which is a less than ideal strategy. To gain a deeper understanding of this matter, Tenaya Therapeutics Inc. (San Francisco, CA, USA) is conducting a multicentre, non-interventional study to investigate the prevalence of pre-existing antibodies to AAV9, which is used for gene therapy, in a population of patients with *PKP2*-associated ARVC (NCT06311708).

Furthermore, natural infection with wild-type AAV also triggers adaptive cell-mediated immune responses targeting the capsid, resulting in the formation of a reservoir of memory cytotoxic T cells that can be reactivated upon vector administration [47]. Not only has T cell reactivity to the AAV capsid been associated with apparent deficiencies in transgene expression [47], but it has also been linked to fatal outcomes. In 2022, Novartis reported that two children with spinal muscular atrophy (SMA) died from acute liver failure attributed to capsid-specific cell-mediated immune responses following treatment with Zolgensma, the first AAV-based gene therapy approved by the FDA for treating SMA [50].

Even in cases where there is no prior exposure to AAV, patients will eventually develop adaptive immune responses after initial treatment. This complicates matters, as administering the same AAV serotype again becomes difficult. This limitation to a single administration raises concerns about achieving the required dosage for optimal transduction in the target organ to ensure durable transgene expression. Striking a delicate balance between the need for single-dose therapeutic efficacy and the potential for excessive innate and humoral immune reactions becomes imperative [51]. Moreover, the unique diversity of the human immune system and its response to high-dose AAV remain poorly modelled in preclinical animal studies. Therefore, crucial insights into these dynamics can only be obtained through human AAV gene therapy clinical trials.

Presently, there are various pharmacological approaches for modulating the host immune system, particularly when administering AAV systemically. These methods are effective for managing both existing immunity challenges and the need for repeated gene therapy administration. Their goal is to reduce pre-existing immunity and hinder the formation of antibodies by specifically targeting B and T cells. Corticosteroids play a significant role as immunomodulators, exerting broad inhibitory effects on both innate and adaptive immune cells. Their primary mechanism involves reducing the production of pro-inflammatory cytokines, chemokines, and T cells, with a lesser impact on B cells [52]. In the administration protocol for Zolgensma, corticosteroid is initiated prior to vector administration and continued for at least 30 days [53]. Similar protocols are employed for many AAV products currently undergoing clinical development [53]. Although prophylactic corticosteroids have demonstrated efficacy in improving outcomes in certain AAV clinical trials, they have proven ineffective in preventing the loss of transgene expression in others [54]. Moreover, their prolonged use poses risks such as adrenal suppression, osteoporosis, insulin resistance, cushingoid features, neuropsychiatric disturbances, muscle atrophy, and skin atrophy [55].

Consequently, efforts are made to enhance the effectiveness of host immune system modulation and to reduce the duration of steroid therapy by utilising steroid-sparing agents. One approach involves the use of calcineurin inhibitors like cyclosporin or tacrolimus, which operate by inhibiting interleukin (IL)-2 transcription. This inhibition hampers T cell differentiation, survival, and subsequent antibody production, along with the activities of cytotoxic T lymphocytes (CTL) via effector T helper (TH) cells [55]. Similarly, sirolimus (rapamycin) targets the same intracellular immunophilin as tacrolimus; however, the rapamycin/FKPB12 complex inhibits the mammalian target of rapamycin (mTOR), leading to beneficial downstream effects, including the generation of regulatory T cells and suppression of CTL and TH activation and, at higher doses, B cell proliferation and differentiation [56]. B cell suppression can also be achieved through agents like rituximab, a monoclonal antibody targeting the CD20 antigen, which induces B cell apoptosis or mediates cytotoxicity through humoral or complement pathways [57]. Ongoing investigations into intramuscular rAAV9 re-administration for Pompe disease (NCT02240407) [58] involve attenuating T and B cell responses with rapamycin and rituximab, respectively, showing promising preliminary results in preventing the formation of anti-capsid and anti-transgene antibodies, with the aim of enabling rAAV re-administration and maintaining effectiveness across different underlying mutations [59]. In the context of ARVC clinical trials, an RP-A601 gene therapy trial for *PKP2*-ARVC is planning to employ rituximab, sirolimus, and prednisone to minimise the risk of early complement-mediated and other innate immune-mediated side effects, as well as to reduce the dosage and duration of steroid treatment [60].

## 5. Conclusions

The progress achieved in gene therapy for ARVC, especially the promising outcomes observed in preclinical studies, marks a significant shift towards a new era in treatments for cardiac genetic disorders. While acknowledging the ground-breaking nature of these preclinical advancements, it is crucial to recognise the challenges associated with translating such success into effective clinical applications. Further research is still needed, particularly in developing effective and safe vectors and delivery methods. These advancements are imperative before gene therapy can be established as a primary treatment option for ARVC patients. In the near future, initiating phase 1 clinical trials involving ARVC patients can offer valuable insights that contribute to a deeper understanding of efficacy, safety, potential toxicity, and immunogenicity, and to the ability to address specific disease characteristics.

## Data Availability

No new data were created.
